# Serum Exhibition and Serum Rashes

**Published:** 1909-05-29

**Authors:** James Dundas


### The General Practitioner's Column.

[Contributions to this Column are invited, and if accepted will be paid for.]

### SERUM EXHIBITION AND SERUM RASHES.

By JAMES DUNDAS, M.B., D.P.H.

The use of sera has now become an essential part of modern therapeutics. Diphtheria, scarlet fever, erysipelas, and puerperal fever are all treated with sera, and in recent years vaccine inoculation for tuberculosis and staphylococcic skin affections has become a routine practice. The exhibition of these substances, while not difficult, is not so simple as mere hypodermic medication. Opsonic investigation has shown us how to control tuberculin treatment and the principles of its action, but much remains unknown or uncertain. "Accidents" in the shape of abscesses and other well-defined disturbances frequently occur, and may be alarming. With certain precautions many of these "accidents" may be avoided.

### The Technique of Injection.

Perhaps the most commonly used are the anti-diphtheritic and anti-streptococcic sera. Each of these is injected in fairly large amounts, so that the practitioner should possess a fairly large syringe, of 10 c.c. capacity at least, and keep it for this purpose alone. These syringes are very liable to get out of order, but careful attention will prevent this, and, as a dirty syringe or needle is always the source of abscess formation, too much care cannot be devoted to them. It is a sound rule after use to pull out the piston to its full extent, manœuvre it until the nut on it engages in the square at the top of the barrel, and by a turn or two of the piston loosen the head of the plunger. The syringe is now ready to be sterilised. Boiling of syringe and needle is the only sound method. The syringe may be then laid aside in its case until required, when the plunger should be tightened up.

Before use it is sufficient to run the syringe through two carbolic solutions—1-20 and 1-80—and then several times through sterile water with the aim of removing the last traces of carbolic. As there is already a certain percentage of carbolic in the serum for preserving purposes, it is inadvisable to add to that amount. The syringe is now ready to be filled. This is most easily done through the wide-bored filler that many makers supply with their instruments. It is removed and the needle affixed, care being taken to expel all air. The site of injection is largely a matter of personal taste. Some prefer to inject between the shoulders, the idea being that the skin is less sensitive there, and that the patient is unable to see what is going on. Objections to this are the comparatively small amount of loose tissue, and that the patient lies on the puncture wound. The writer's invariable practice is to put the patient on the side and stand behind him out of his field of vision. The point chosen lies in the flank half-way between the ribs and the pelvis. The loose skin is pinched up and the needle inserted to its full extent boldly, but without violence. The advantages of this position are that no pressure is exerted on the site of injection, the patient sees little of what is going on, and is easily held by an assistant exerting pressure on the great trochanter with one hand and controlling the hands with the other. It is a mistake to inject over a rib: the convexity of the rib may snap the needle if the child struggles. On one occasion the writer made an injection into the mamma in a case of malignant endocarditis, in which, on account of collapse and for other reasons, it was not deemed wise even to roll the patient over. Next morning the breast was large, somewhat tense and tender, and the skin was very dark in colour. Fortunately, in a few days the condition disappeared. In several hundreds of injections that is the only accident experienced.

The serum should be injected slowly, as the pain of the operation depends on the separation of the tissues; and concurrently the needle should be withdrawn. On removal of the needle the nurse or assistant should immediately apply a pad of lint and collodion, as the serum tends to leak from the skin puncture. Should the syringe be too small for the quantity of serum to be injected, it is unnecessary to remove the needle. It suffices to withdraw the syringe from the needle, recharge it, expel air, and reinsert it in the needle. This last must be done securely. It cannot be too strongly insisted that abscesses are due to sepsis, and that careful attention to Listerian principles will obviate them.

### Rashes and Other Sequelæ.

Certain sequelæ of serum exhibition may be expected, and in America one or two deaths have been put down to them. Their causes are not fully understood at present, but it seems evident that they depend on the injection of too much serum for the amount of toxin requiring to be neutralised; and often on a previous injection some days before. This means that too many anti-bodies are produced, and they are, according to V. Pirquet, the source of the trouble. As we have no means, save the rough clinical one, of estimating the degree of toxicity, we are bound at times to overdose the patient. Thus prophylactic inoculations into diphtheria contacts may produce the signs of the so-called "serum disease," and several cases of this type have been reported recently by practitioners who had injected themselves.

Another well-established fact is that repeated doses are more liable to produce symptoms than solitary ones, especially in the case of diphtheria, if the second injection is made ten days to a fortnight after the first. The explanation, of course, is that the anti-bodies, which have taken that time to be produced, come into violent collision with the newly injected serum. The result may be surprising and very alarming unless one is prepared for it, as within half an hour, or even ten minutes, the patient may be sick and ill, and have a general rash.

The first sign is enlargement of glands to about the size of a split pea in the groin and axilla on the side of injection. These have usually to be looked for, as the patient rarely complains of them. Within a few hours a rash appears, generally round the site of injection, and most usually of urticarial type. This rash may spread over the entire body; or give place to a morbilliform, or, less frequently, a scarlatiniform, eruption which becomes general. The first rash is almost always urticarial; a second may not appear. None of these rashes is persistent. They come and go, and vary in intensity. Their favourite sites are round the needle puncture and on the extensor surfaces of the large joints. On the second day the glands may be the size of a bean, and may possibly be felt in both groins and axillæ. Localised patches of œdema occur. There may be albumen in the urine; whether this is due to the serum is a moot point, the time of onset being late for a diphtheritic albuminuria.

Joint pains and headache are common, but by no means constant, and nothing seems to be very efficacious in relieving them. The small joints of the fingers and wrists, the ankles, knees, and neck are the common situations. "Joint pains" most nearly describes these phenomena. A thorough-going arthritis is not so common.

The whole disturbance lasts four or five days, and is comparatively mild: only in a few cases is there any rise of temperature. As these symptoms, or some of them, occur in, roughly, 50 per cent. of cases receiving serum, it is as well to safeguard oneself by warning parents of their probable occurrence. Doubt may be entertained of the diagnosis when those rashes appear, and one may be accused of adding measles or scarlet fever to the ills of the patient. Their evanescent character and the absence of catarrhal conditions in the nose and throat serve to differentiate them from these exanthems. The joint pains might suggest acute rheumatism, but the presence of the rash and the absence of a visible acute arthritis serve to clear up the diagnosis.

In scarlet fever anti-streptococcic serum is now being largely used in selected cases. These are the early and intensely septic cases with marked rhinorrhœas, dirty throats and mouths. The degree of toxicity is great, and there is no sign of reaction. In such cases, when, perhaps, 60 c.c. of serum have been exhibited, somnolence ensues and the patient is lethargic, possibly for weeks. Associated with this one finds a general lymphatic enlargement which rarely suppurates. Quite suddenly such cases waken up and begin to take an interest in life. Recovery is slow. There may be joint pains, though they are not evident during the sleepy stage. Rashes are frequent on the extensor surfaces of the limbs about the joints, and are septic in character. An associated erythematous rash rather indicates the serum as being the cause. These phenomena, if uncomfortable, are not dangerous, and the results of treatment justify one in risking the temporary disturbance.

